# Correlation of Tryptophan Metabolic Pathway with Immune Activation and Chemosensitivity in Patients with Lung Adenocarcinoma

**DOI:** 10.1155/2022/2158525

**Published:** 2022-09-21

**Authors:** Zheng Wang, Danni Liu, Rongjie Yang

**Affiliations:** ^1^Department of Thoracic Surgery, Cancer Hospital of Dalian University of Technology, Liaoning Cancer Hospital & Institute, Shenyang, China; ^2^Department of Biological Information Research, HaploX Biotechnology, Shenzhen, China 518035

## Abstract

Lung adenocarcinoma (LUAD) is the most common type of lung cancer with high malignancy and easy metastasis in the early stage. In this study, we aimed to figure out the role of tryptophan metabolic pathway in LUAD prognosis and treatment. Different molecular subtypes were constructed based on tryptophan metabolism-related genes. Significant prognostic genes and clinical prognostic characteristics, immune infiltration level, and pathway activity in different subtypes were determined by algorithms, such as the least absolute shrinkage and selection operator (Lasso), CIBERSORT, Tumor Immune Dysfunction and Exclusion (TIDE), and gene set enrichment analysis (GSEA). The effect of different gene mutation types on the prognosis of patients with LUAD was explored. The clinical prognosis model was constructed and its reliability was verified. Of the 40 genes in the tryptophan metabolism pathway, 13 had significant prognostic significance. Based on these 13 genes, three molecular subtypes (C1, C2, and C3) were established. Among them, C1 had the worst prognosis and the lowest enrichment score of tryptophan metabolism. At the same time, C1 had the most genetic variation, the highest level of immune infiltration, and significantly activated pathways related to tumor development. The high-risk and low-risk groups had significant differences in prognosis, immune infiltration and pathway enrichment, which was consistent with the results of subtype analysis. Mutation in tryptophan metabolism-related genes leads to abnormal tryptophan metabolism, immune deficiency, and activation of cancer-promoting pathways. This results in high malignancy, poor prognosis, and failure of traditional clinical treatments. Through the establishment of risk score (RS) clinical prognosis model, we determined that RS could reliably predict the prognosis of patients with LUAD.

## 1. Introduction

Lung cancer is one of the most aggressive and rapidly fatal tumor types, accounting for more than 25% of all cancer-related deaths [1]. Based on histological characteristics, it can be divided into non-small-cell lung cancer (NSCLC) and small cell lung cancer (SCLC), which account for 85% and 15% of all cases, respectively [2]. NSCLC further includes lung adenocarcinoma (LUAD), lung squamous cell carcinoma, and large cell carcinoma, where LUAD is the most essential type that accounts for approximately 40% of NSCLC [3]. Although new drugs for lung cancer treatment have been developed, they tend to fail due to the gradual emergence of drug resistance in patients. Key gene mutations and abnormal body metabolism are the genetic factors that regulate the sensitivity of tumors to cell death-inducing factors. Therefore, finding key targets that affect body metabolism may help in developing innovative strategies for the treatment of LUAD [4].

Tryptophan (Trp) is an essential amino acid for the human body. Trp and its metabolites play a key role in a variety of physiological processes, ranging from cell growth and maintenance to coordinating the body's response to environmental and dietary cues [5]. Trp metabolism regulates immunity, neuronal function, and intestinal homeostasis through the kynurenine pathway (KP). More than 95% of free Trp is degraded through KP ([6, 7], Le [8]). The imbalance of Trp and its metabolites is related to a variety of human diseases including depression, schizophrenia, autoimmunity, neurodegeneration, and cancer [9]. In cancer, indoleamine 2,3-dioxygenase 1 (IDO1) and tryptophan 2,3-dioxygenase (TDO) catalyze the first and the rate-limiting step of tryptophan metabolism. Their abnormal activation leads to antitumor immunosuppression, which is related to tumor immune tolerance and poor prognosis of patients and has become a critical target of tumor immunotherapy. In recent years, IDO1 inhibitors have been studied for cancer immunotherapy in clinical trials, usually in combination with other drugs, such as immune checkpoint inhibitors [10]. The increased expression of IDO1 and TDO in malignant tumors leads to tryptophan depletion and accumulation of downstream products. This creates an immunosuppressive microenvironment and enables tumor cells to escape from effective immune response. Additionally, tryptophan depletion can also inhibit mammalian target of rapamycin- (mTOR-) mediated molecular stress response and induce autophagy of T_eff_. The combination of the catabolic metabolites of tryptophan and kynurenine with endogenous aromatic hydrocarbon receptor (AHR) leads to selective differentiation and proliferation of regulatory cells (T_regs_). At the same time, it prevents the maturation of T helper 17 cell (Th17) and thus inhibits the infiltration of dendritic cells and the immune response of cytotoxic T cells (T_c_) [11]. In addition to the immune response induced by tryptophan and kynurenine, the production of quinolinic acid, 3-hydroxykynurenine, and other metabolites suppresses the transformation of macrophages and inhibits the proliferation and function of natural killer (NK) cells. These pathways jointly mediate local and/or systemic immune inhibition and promote the survival and metastasis of tumor cells [12]. Immunotherapy can recognize and eliminate tumor cells by restoring or even activating the natural immune system of cancer patients. The tryptophan metabolism appears to be a key target of tumor immunotherapy. However, there are limited reports on the potential biological effects of Trp metabolic pathway in LUAD. Therefore, the aim of this study was to determine the effect of Trp metabolism-related genes on the prognosis of patients with LUAD and predict their outcomes.

## 2. Materials and Methods

### 2.1. Downloading and Filtering the Training Set and Verification Set Data

For the training set data, LUAD data was downloaded from The Cancer Genome Atlas (TCGA) database using TCGA (https://www.cancer.gov/) GDC API. The samples lacking clinical data were removed, and the Ensembl number was converted to the gene symbol. For the duplicate gene symbol, the middle value was taken. Finally, 565 primary tumor samples having mutation data, copy number data, and transcriptome data were included in the training set (TCGA-LUAD). In the Gene Expression Omnibus (GEO) database, GSE31210 and GSE72094 were selected as independent validation set data. After downloading the corresponding GPL file, the probes corresponding to multiple genes were deleted. When multiple probes matched the same gene, the median value was taken as the gene expression level. After selection, 226 samples were obtained from GSE31210 and 398 were obtained from GSE72094.

### 2.2. Sources and Molecular Subtypes of Tryptophan Metabolism-Related Genes

The tryptophan metabolism-related genes were identified from the tryptophan metabolism pathway “KEGG_tryptophan_metabolism” in the Molecular Signatures Database (MSigDB, https://www.GSEa-msigdb.org/GSEa/msigdbs/), which contained 40 tryptophan metabolism-related genes. Among these, the genes related to tryptophan metabolism with prognostic significance were identified by univariate Cox regression. According to the expression profiles of genes with prognostic significance, a consistent cluster was constructed. ConsensusClusterPlus [13] used “km” algorithm and “1-Spearman correlation” as distance measurement and performed 500 bootstraps. Each bootstrap process included 80% of patients in the training set. The number of clusters was set as 2 to 10. The best classification was determined by calculating the consistency matrix and the consistency cumulative distribution function, and the molecular subtypes of the samples were obtained.

### 2.3. Tumor Microenvironment (TME) Analysis

Immune cells play an indispensable role in the TME. The CIBERSORT algorithm (https://cibersort.stanford.edu/) was used to analyze the difference in the degree of immune cell infiltration of 22 kinds of immune cells in the TCGA-LUAD. Additionally, the ESTIMATE algorithm [14] was used to calculate the immune score and matrix score. Abnormal expression and function of immune checkpoint molecules are one of the main causes of tumor. The transcription levels of a large number of immune checkpoint molecules were analyzed in different molecular subtypes based on the expression data of TCGA-LUAD. The Tumor Immune Dysfunction and Exclusion (TIDE) algorithm was used to calculate the difference in immunotherapy sensitivity of the different subtypes. The higher the TIDE prediction score, the higher the possibility of immune escape, which suggests that patients were less likely to benefit from immunotherapy.

### 2.4. Functional Pathway Analysis

Gene set variation analysis (GSVA) is an algorithm to explore the relationship between samples and pathways and performs unsupervised clustering on samples. To explore the impact of tryptophan metabolism gene mutations on the samples, these genes were divided into two groups based on whether they were mutated for pathway difference analysis. At the same time, gene set enrichment analysis (GSEA) was performed among the different subtypes based on the candidate gene sets in the Hallmark database [15], and whether there was a significant difference in pathways (false discovery rate (FDR) < 0.05) was analyzed.

### 2.5. Constructing Risk Model by Combining Differentially Expressed Genes (DEGs) among Molecular Subtypes with Prognosis Data

The DEGs of C1 vs. non-C1, C2 vs. non-C2, and C3 vs. non-C3 were analyzed by using the data of TCGA expression profile using “limma” package [16]. The threshold was set as FDR < 0.05 and |log_2_ fold change| > 1.5. The differential genes with prognostic significance were identified by univariate Cox regression combined with clinical data (*P* value < 0.01). Next, the least absolute shrinkage and selection operator (Lasso) regression was performed using “glmnet” R package [17], and stepwise multivariate regression analysis was performed using “MASS” R package [18]. The final genes were regarded as the key prognostic genes of tryptophan metabolism. At the same time, each TCGA-LUAD sample was given a risk score (RS) using the following formula: RS = *Σ* (*β*_*i*_ × Exp_*i*_). Exp_*i*_ is referred to as the expression level of key prognostic genes of tryptophan metabolism, and *β*_*i*_ is referred to as the Cox regression coefficient of the corresponding gene. The samples were divided into high- and low-risk groups based on the threshold value “0.” For the two groups, the Kaplan-Meier method was used to draw the survival curve for prognostic analysis, and log rank test was used to determine the significance of the difference.

### 2.6. Prediction of the Response to Immunotherapy and Chemotherapy

Tumor Immune Dysfunction and Exclusion (TIDE) algorithm [19] was utilized to assess the therapeutic response to immune checkpoint inhibitors. A TIDE score was estimated by the enrichment of immunosuppressive cells, T cell dysfunction, and exclusion. A higher TIDE score represents less responsive to immunotherapy and a higher possibility of immune escape. The predictive response to chemotherapeutic drugs was estimated by pRRophetic R package [20].

### 2.7. Statistical Analysis

All statistical methods used in this study were operated using the R software (version 4.0, https://www.r-project.org/). A *P* value of <0.05 was regarded as statistically significant.

## 3. Results

### 3.1. A Large Number of Mutations and Transcriptional Differences in Tryptophan Metabolism-Related Genes in TCGA-LUAD

**Figure 1:**
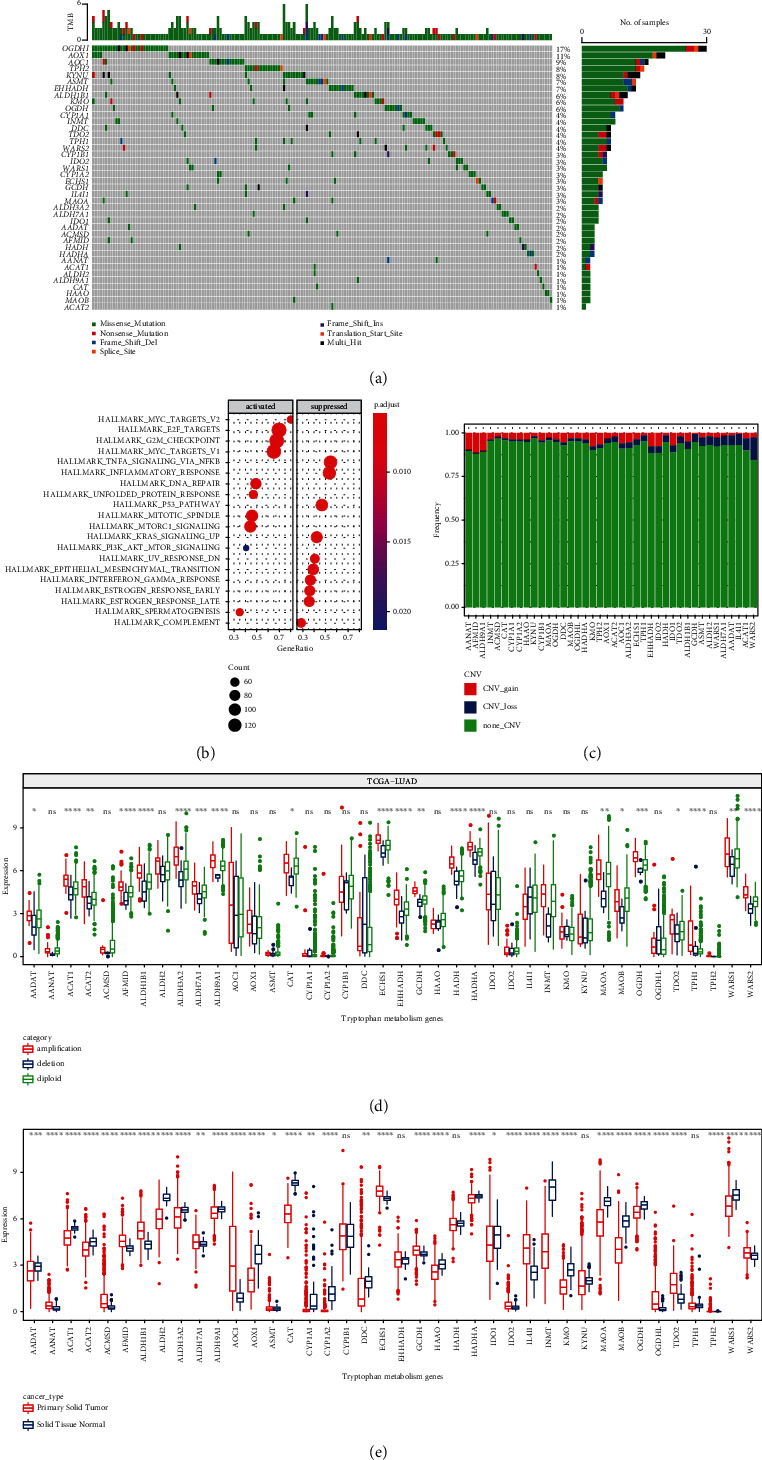
Mutation frequency and transcription level of tryptophan metabolism-related genes in TCGA-LUAD. (a) Total mutation frequency of tryptophan metabolism-related genes in TCGA-LUAD. (b) GSEA between mutant and nonmutant groups of tryptophan metabolism-related genes in TCGA-LUAD. Pathways with normalized enrichment score (NES) < 0 defined as the inhibited group and NES > 0 as the activated group. (c) Copy number variation map of tryptophan metabolism genes. (d) Difference in transcription levels of tryptophan metabolism genes among different copy number variants. (e) Transcriptional differences in tryptophan metabolizing genes between tumor and normal tissues. ns: not significant. ^∗^*P* < 0.05, ^∗∗^*P* < 0.01, ^∗∗∗^*P* < 0.001, and ^∗∗∗∗^*P* < 0.0001.

Firstly, 40 tryptophan metabolism-related genes were obtained from MSigDB (Table S1). The mutation frequency was calculated based on the mutation data in TCGA-LUAD. The results showed that a total of 181 samples had tumor mutation burden (TMB), of which OGDHL gene had the highest mutation frequency, followed by AOX1 and AOC1. Most of the gene mutations were missense mutations, in addition to nonsense mutations and transcription start site mutations (Figure 1(a)). Meanwhile, GSVA was conducted to explore the differential pathways in mutant and wild-type (tryptophan metabolism-related genes) groups. It was found that the pathways significantly activated in the mutant group included MYC targets V1/V2, E2F targets, and G2M checkpoint, whereas the pathways significantly inhibited in the mutant group included TNF*α* signaling via NF-*κ*B and inflammatory response (Figure 1(b)).

The copy number variations (CNVs) of 40 tryptophan metabolism-related genes were explored in the samples with tryptophan gene mutations. The results showed that CNVs appeared in all samples. AANAT had the maximum copy number amplification frequency and WARS2 had the least copy number deletion (Figure 1(c)).

Additionally, TCGA-LUAD samples were divided into three groups according to CNVs, including CNV gain (amplification group), CNV loss (deletion group), and no significant change in CNV (diploid group). According to the grouping, more than half of the genes had significant differences. The transcription level of tryptophan metabolism genes in the CNV amplification group was significantly higher than that in the CNV deletion group. This suggests that CNV plays a critical role in the tryptophan metabolism pathway (Figure 1(d)). We showed the expression levels of tryptophan metabolism-related genes in tumor and normal samples. There were significant differences in most genes, suggesting the correlation between tryptophan metabolism and tumor (Figure 1(e)).

### 3.2. Molecular Typing Based on Tryptophan Metabolism-Related Genes

**Figure 2:**
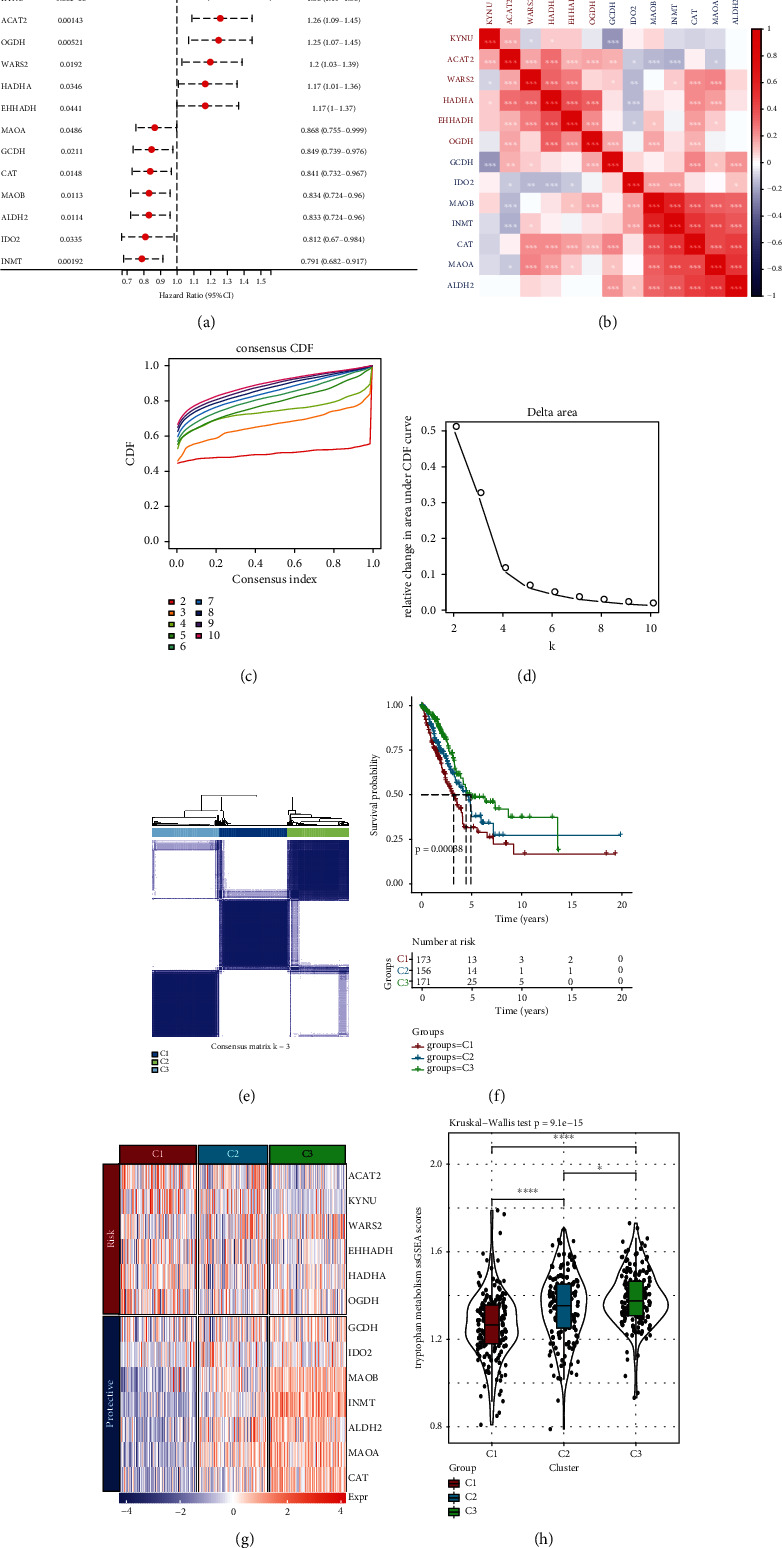
Construction of molecular subtypes based on tryptophan metabolism genes. (a) Cox regression analysis showed tryptophan metabolism genes with prognostic significance. (b) Heat map of the correlation between tryptophan metabolism genes and prognostic genes. (c) CDF curve was constructed based on TCGA-LUAD. (d) CDF delta area curve was constructed based on TCGA-LUAD. (e) Sample clustering heat map when consensus *k* = 3. (f) Survival curves of three molecular subtypes. (g) Expression heat map of tryptophan metabolism genes with prognostic significance in different subtypes. (h) Difference in “tryptophan metabolism ssGSEA scores” among different molecular subtypes in TCGA-LUAD cohort. ^∗^*P* < 0.05 and ^∗∗∗∗^*P* < 0.0001.

**Figure 3:**
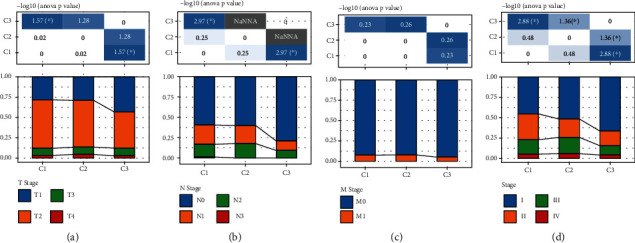
Distribution of clinical information in different molecular subtypes. (a–c) TNM staging. (d) Pathological staging. ^∗^*P* < 0.05. NaNNA represents no test was performed as a severe imbalance of the distribution in two groups.

In order to identify tryptophan metabolism-related genes that have prognostic significance, univariate Cox regression was performed on 40 tryptophan metabolism-related genes based on the clinical data of TCGA-LUAD. The results showed that 13 genes had significant prognostic significance, of which 6 were risk genes and 7 were protective genes (Figure 2(a)). Based on the expression profile data, there was positive correlation among the six protective genes and negative correlation between protective genes and risk genes (Figure 2(b)). After that, molecular typing was constructed based on 13 prognostic genes, and the optimal number of clusters determined by the cumulative distribution function (CDF) was 3. When *k* = 3, the clustering results were relatively stable (Figures 2(c)–2(e)). The different prognosis results among the three molecular subtypes were analyzed based on the clinical data of TCGA-LUAD. It was found that C1 had the worst prognosis while C3 had the best prognosis (Figure 2(f)). In addition, the transcription level of protective genes was the highest in C3 and that of risk genes was the highest in C1. This suggests that a decline in the transcription level of protective genes and a rise in the transcription level of risk genes would have an adverse impact on the prognosis of patients (Figure 2(g)). In addition, the “tryptophan metabolism ssGSEA scores” of different subtypes were calculated. The results showed that C1 scored the lowest, whereas C3 scored the highest. This indicates that the activation of tryptophan metabolic pathway had a positive significance on the prognosis of patients (Figure 2(h)). Meanwhile, according to the distribution of TNM stage and pathological stage in different subtypes, tumor progression was low in C3, while high in C1 (Figures 3(a)–3(d)).

### 3.3. Mutation Differences among Molecular Subtypes

**Figure 4:**
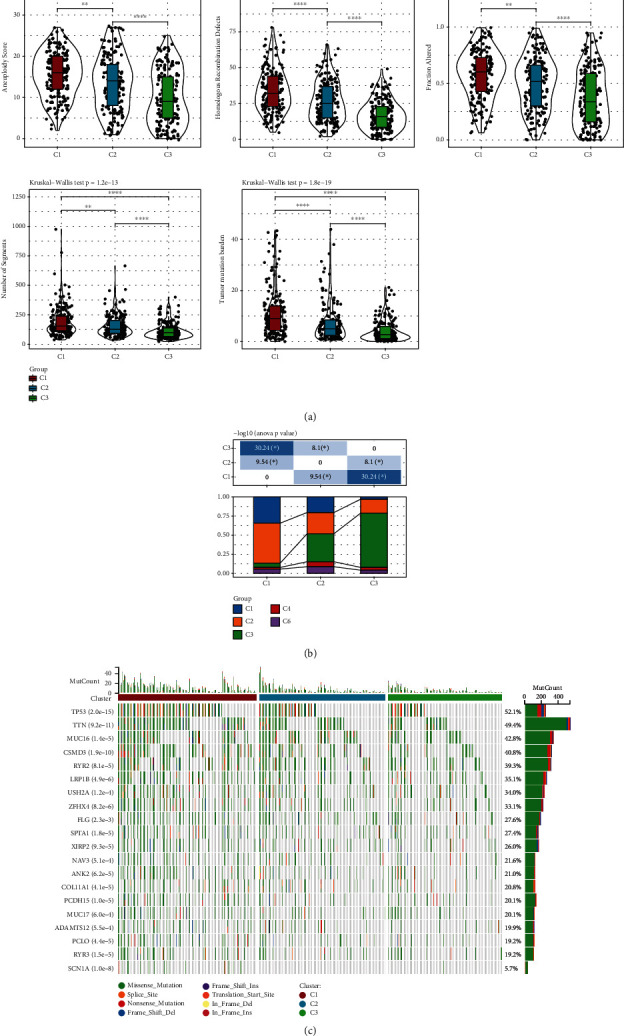
Frequency distribution of different subtypes in TCGA-LUAD. (a) Distribution of different mutation types in different subtypes. (b) Comparison of three molecular subtypes and immune molecular subtypes. (c) Distribution map of somatic mutation frequency in three subtypes. ^∗^*P* < 0.05, ^∗∗^*P* < 0.01, and ^∗∗∗∗^*P* < 0.0001.

In order to explore the mutation differences among the molecular subtypes, the molecular characteristics of TCGA-LUAD were obtained from a previous pan-cancer study [21]. It was evident that C1 with the worst prognosis had the highest aneuploidy score, homologous recombination defects, fraction altered, number of segments, and TMB. This indicates that the higher the mutation frequency, worse is the prognosis of patients with LUAD (Figure 4(a)). According to the pan-cancer research, five different molecular subtypes were constructed based on 160 immune signatures, and the reported immune subtype C3 (inflammatory) had the best prognosis. Additionally, the expression levels of Th17 and Th1 genes were higher, and the proliferation degree of tumor cells and CNV level were lower in the immune subtype C3 (inflammatory) than other subtypes. Interestingly, the C3 defined in this study was similar to the immune subtype C3, and our results were consistent with previous ones (Figure 4(b)). Based on the mutation data in TCGA-LUAD, the mutation frequency of some tumor suppressor genes, such as TP53, was much higher in C1 than other subtypes. This again verifies the impact of mutation frequency on the prognosis of TCGA-LUAD (Figure 4(c)).

### 3.4. Differences in Immune Infiltration and Sensitivity to Immunotherapy/Chemotherapy among Molecular Subtypes

**Figure 5:**
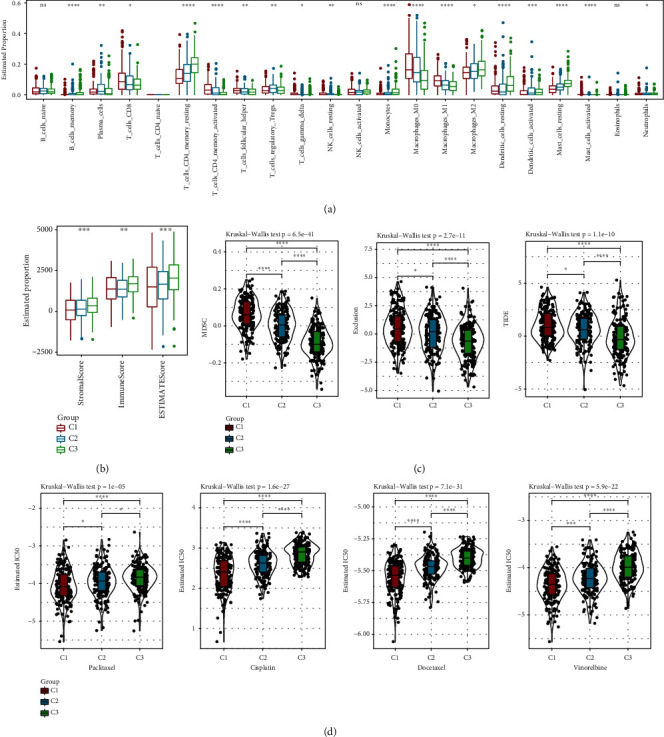
Difference in immune infiltration between different subtypes. (a) Difference in infiltration of 22 kinds of immune cells between different subtypes. No statistical test was performed on naïve CD4 T cells as almost no detection of their enrichment. (b) Difference in immune infiltration between different subtypes was calculated based on the ESTIMATE algorithm. (c) Difference in infiltration of different immune cells was calculated based on TIDE algorithm. (d) Box plot of IC50 of paclitaxel, cisplatin, docetaxel, and vinorelbine in different subtype groups.

Immune microenvironment is an indispensable component of tumorigenesis and development. The infiltration scores of 22 different immune cells were calculated using CIBERPORT algorithm based on the expression profile data of TCGA-LUAD. 18 of 22 immune cells were differentially distributed in three subtypes such as CD8 T cells, resting memory CD4 T cells, M0 macrophages, M1 macrophages, and M2 macrophages (Figure 5(a)). At the same time, the stromal score and immune score were calculated by ESTIMATE. Both the stromal and immune score were much higher in C3 than in the other two subtypes (Figure 5(b)).

In addition, infiltration differences in three immunosuppressive cells were calculated in different subtypes. Firstly, the infiltration level of myeloid-derived suppressor cells (MDSCs) was the lowest in the C3 subtype. In addition, T cell exclusion and TIDE exhibited the same trend as MDSCs. This indicates that C1 with the highest TIDE score had a greater possibility of escape from immunotherapy, which might be related to the high infiltration of MDSCs (Figure 5(c)). The drug sensitivity of different molecular subtypes to different chemotherapeutic drugs, including paclitaxel, cisplatin, docetaxel, and vinorelbine, was analyzed. The results indicate that C1 had the highest sensitivity to these four chemotherapeutic drugs (Figure 5(d)).

### 3.5. Pathway Differences between Molecular Subtypes

All candidate gene sets were enriched and analyzed by GSEA to explore the pathways between different molecular subtypes. There were 38 pathways that finally met the threshold. In C1, 11 pathways were significantly inhibited and 16 pathways were significantly activated. The pathways significantly activated in C1 were cell cycle-related pathways, namely, G2M checkpoint, E2F targets, and MYC targets V1/V2. These pathways were inhibited in C3, whereas the pathways significantly inhibited in C1 were bile acid metabolism, coagulation, and early estrogen response. These pathways were activated in C3. In C2, only one pathway was activated, namely, glycolysis, whereas 20 pathways were significantly inhibited, which include interferon gamma response, allograft rejection, and IL2-STAT5 signaling (Supplement Figure 1A).

### 3.6. Identifying Key Genes of Tryptophan Metabolism and Establishing a Prognosis Model

**Figure 6:**
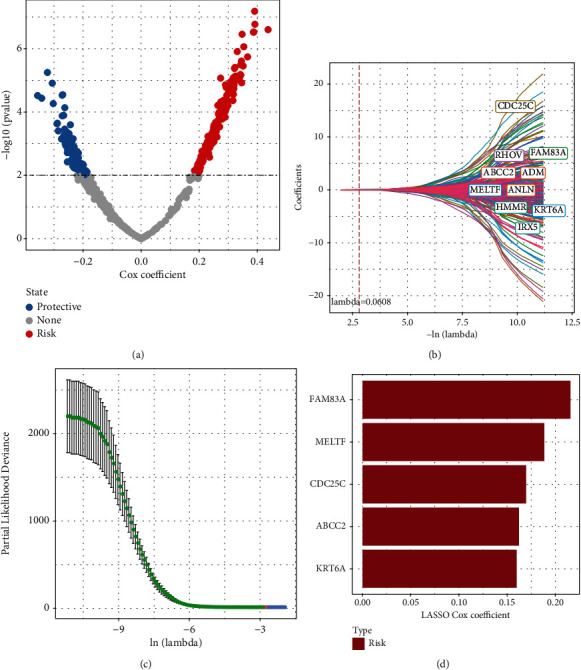
Identification of key genes of tryptophan metabolism phenotype. (a) The volcano map shows 272 candidate key genes. (b) The trajectory of each independent variable with lambda. (c) Confidence interval under lambda. (d) Five key genes of tryptophan metabolism phenotype were identified.

Given that three subtypes had differential prognosis and molecular features, we then analyzed the DEGs among them by analyzing C1 vs. non-C1, C2 vs. non-C2, and C3 vs. non-C3. A total of 562 DEGs were identified. Of these, 272 genes with great impact on prognosis were identified by univariate Cox regression, including 154 risk genes and 118 protective genes (Figure 6(a)). After that, the 272 prognostic genes were identified by lasso regression model. When *λ* = 0.0608, the model reached the optimal level (Figures 6(b) and 6(c)). Therefore, 10 genes when *λ* = 0.0608 were selected as the target genes for the next step. Through stepwise multifactor regression analysis, the model had sufficient fit. Finally, five genes were identified as key prognostic genes of tryptophan metabolism, including FAM83A, MELTF, CDC25C, ABCC2, and KRT6A (Figure 6(d)).

**Figure 7:**
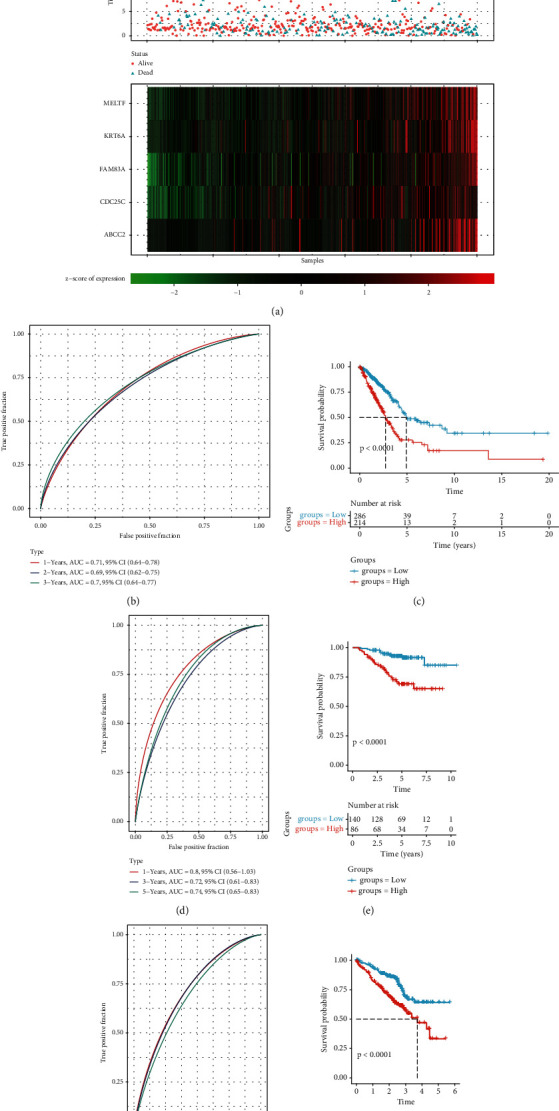
Establishment and validation of the clinical prognosis model. (a) Distribution of risk factors of 5 key genes. (b) ROC curve verifies the reliability of the model. (c) Survival curves under different RS groups. (d–g) ROC curve and survival curve of the two validation sets show the accuracy of the clinical prognosis model.

Based on our prognostic model formula, each sample in TCGA-LUAD was scored and the risk score was normalized to *z*-score. As shown in Figure 7(a), as the RS increased, the prognosis of patients became worse. The expression of the five key prognostic genes of tryptophan metabolism also increased significantly as the RS increases. In addition, a time-dependent ROC prognostic analysis was conducted on RS to assess the effectiveness of the model in predicting the prognostic outcomes in 1, 3, and 5 years. The results showed that the model had strong predictive ability (Figure 7(b)). RS equal to 0 was taken as the dividing line. Samples > 0 were classified as the RS-high group, while samples < 0 were classified as the RS-low group. By analyzing the difference in prognosis between the two groups, it was found that the prognosis of the RS-high group was significantly worse than that of the RS-low group (Figure 7(c), *P* value < 0.0001). In addition, two independent datasets from GEO database were used as the validation set. The same prognosis model as TCGA-LUAD was carried out with the relevant data of the validation set to test the stability of the clinical prognosis prediction model based on tryptophan metabolism gene. The results in the validation set showed that the model was very stable (Figures 7(d)–7(g)).

### 3.7. RS Distribution in Different Clinical Stages and Molecular Subtypes

**Figure 8:**
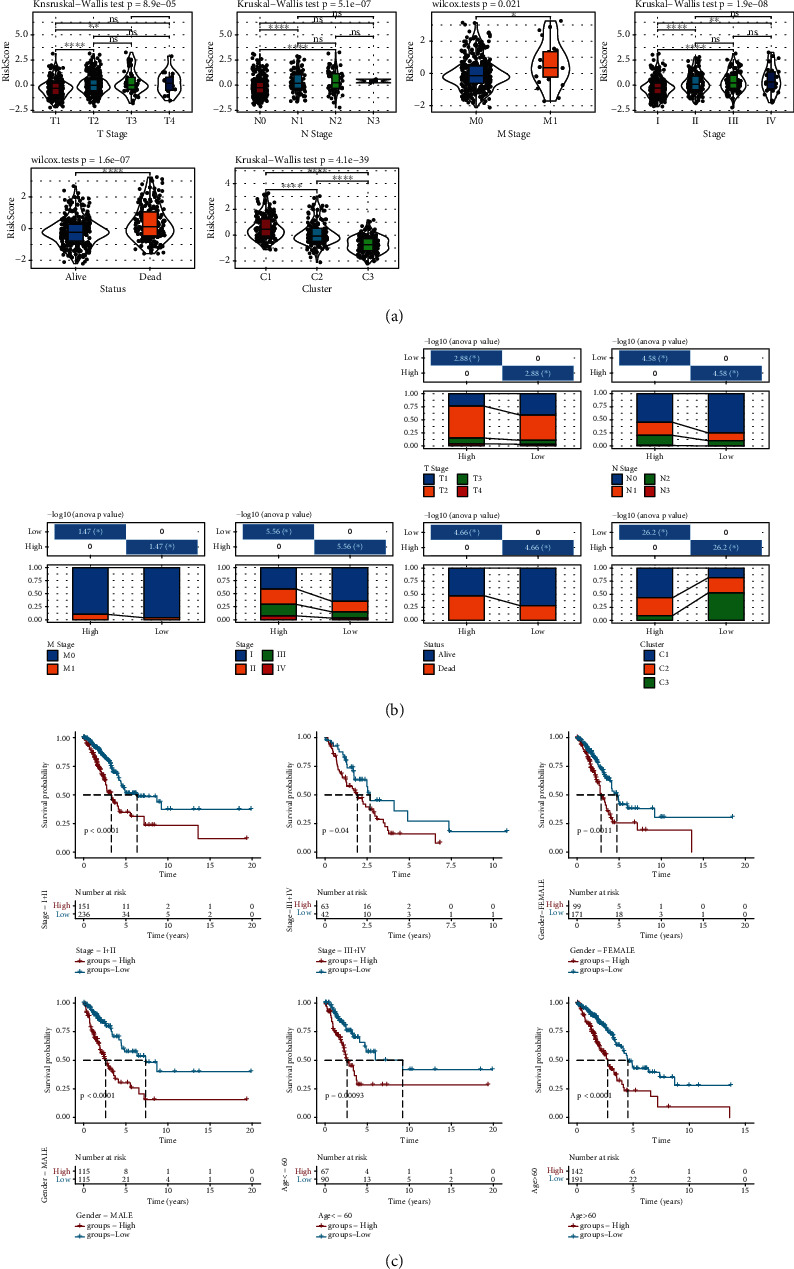
Distribution of clinical characteristics in different RS groups. (a) Differences among clinicopathological groups according to RS in TCGA-LUAD. (b) Clinicopathological features between RS groups in TCGA-LUAD. (c) The survival curves of different clinical features according to RS. ns: not significant. ^∗^*P* < 0.05, ^∗∗^*P* < 0.01, and ^∗∗∗∗^*P* < 0.0001.

According to the clinical data of TCGA-LUAD and the RS data of the clinical prognosis prediction model of tryptophan metabolism gene, the RS gradually increased with the development of tumor, and the prognosis worsened. The RS of men was significantly higher than that of women, and the RS of C3 was much lower than that of C1 and C2, which is consistent with our previous analysis. C3 subtypes had the best prognosis and more women (Figures 8(a) and 8(b)). We compared whether there were prognostic differences between RS groups in different clinical pathological feature groups. The results showed that the prognostic outcomes of the RS-high group were worse than those of the RS-low group in different clinical subgroups, which once again proved the reliability of our model (Figure 8(c)). In addition, we found that RS and tryptophan metabolism ssGSEA score showed a negative correlation trend. Combined with the previous studies, in this study we inferred that inhibition of tryptophan metabolism would lead to a rise in RS and ultimately worsen prognosis (Supplement Figure 1B).

### 3.8. Predictive Responses of Two Risk Groups to Immunotherapy and Chemotherapy

**Figure 9:**
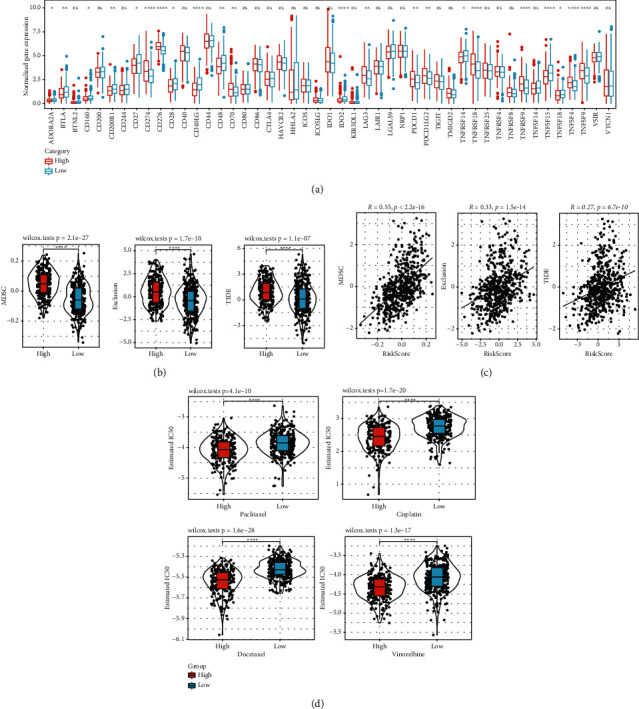
Predictive response of two risk groups to immunotherapy and chemotherapy. (a) The expression of immune checkpoints in two risk groups. (b) Analysis of immune cell scores in RS groups. (c) Correlation analysis between scores of different immune cells and RS. (d) IC50 box diagram of paclitaxel, cisplatin, docetaxel, and vinorelbine in different RS groups. ns: not significant. ^∗^*P* < 0.05, ^∗∗^*P* < 0.01, ^∗∗∗^*P* < 0.001, and ^∗∗∗∗^*P* < 0.0001.

The MDSC score of the RS-high group was higher than that of the RS-low group. According to previous analysis, the immune infiltration level of C3 was much higher than that of other subtypes, and C3 accounted for a very small proportion in the RS-high group. This indicates that immune infiltration in the RS-high group was significantly inhibited. Subsequently, we evaluated the response of two risk groups to immunotherapy. Immune checkpoint blockade is a promising immunotherapy for treating metastatic cancer patients, and its efficiency is associated with the expression of immune checkpoints. We compared the expression of immune checkpoints in two risk groups and observed that 22 of 47 immune checkpoints including CD274 (PD-L1) and PDCD1 (PD-1) were differentially expressed between two risk groups (Figure 9(a)). Additionally, the T cell exclusion score was significantly high in the RS-high group, indicating that the immune level in the RS-high group was significantly low. The scores of MDSCs and T cell exclusion showed a significant positive correlation with RS (Figures 9(b) and 9(c)). After assessing the responsiveness of the RS group to the four traditional chemotherapy drugs, we found that the RS-high group was more sensitive to these drugs. This indicates that the effect of these four chemotherapy drugs was better in the RS-high group than in the RS-low group (Figure 9(d)).

### 3.9. Improvement of Prognosis Model and Survival Prediction by RS Combined with Clinical Characteristics

**Figure 10:**
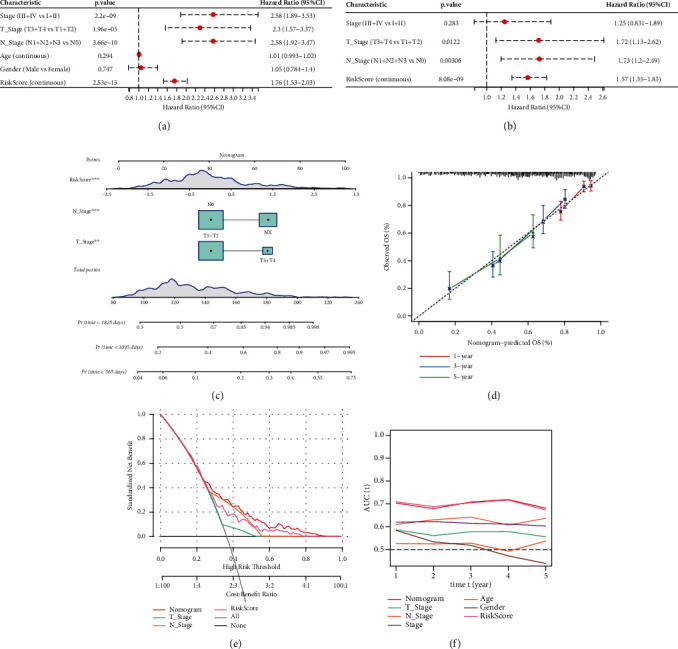
RS was identified as a reliable variable for predicting patient survival. (a, b) Univariate/multivariate Cox regression analysis for different clinical variables and RS. (c) A nomogram model based on different clinical variables and RS. (d) Calibration curve of nomograph for 1, 3, and 5 years. (e) Decision curve of nomograph. (f) Compared with other clinicopathological features, nomogram shows that RS has the strongest ability to predict survival.

The influence of different variables on prognosis was analyzed by univariate/multivariate Cox regression. Both forest map and nomogram showed that TNM and pathological stage were significant prognostic factors, and RS was a significant risk prognostic factor (hazard ratio, HR = 1.76 (1.53-2.03), *P* value < 0.0001, Figures 10(a)–10(c)). Then, a calibration curve was constructed to assess the model's evaluation of the prediction effect of the actual results. It showed that the predicted calibration curve of the three calibration points at 1, 3, and 5 years was close to the standard curve. This indicates that the fitting between the actual probability and the probability predicted by the model was very good (Figure 10(d)). Through the decision curve analysis (DCA) to evaluate the reliability of the model, we found that the benefits of RS and nomogram were significantly higher than the extreme curve. Compared with other clinicopathological features, nomogram and RS showed strong ability to predict survival (Figures 10(e) and 10(f)).

## 4. Discussion

Although groups of immunotherapy clinical trials have made promising outcomes for advanced LUAD treatment, still a large fraction of LUAD patients benefit little from the immunotherapy. The paves to find targeted and personalized therapy should never been stopped. Tryptophan catabolism is considered as a potential therapeutic target for cancer treatment in the recent years [22]. In this study, we obtained 40 tryptophan metabolism-related genes from the MSigDB. Of these, 13 genes significantly correlated with the prognosis of LUAD, suggesting that these genes might play an essential role in the tryptophan metabolism pathway. Then, three molecular subtypes were constructed based on 13 tryptophan metabolism-related prognostic genes. Six risk genes were significantly overexpressed in C1, whereas seven protective genes were significantly overexpressed in C3, which was consistent with their prognostic outcomes that C1 had the worst prognosis and C3 had the longest overall survival. This not only showed the rationality of the molecular typing model but also that these genes affected the prognosis of patients through tryptophan metabolic pathways. After observing the parameters related to gene mutation such as TMB, we found that mutation frequency in C3 was much lower than that in other subtypes. This indicates that gene mutation frequency is one of the factors that affect prognosis of patients. Combined with the previous ssGSEA score of tryptophan metabolism, we found that the ssGSEA score of tryptophan metabolism had a positive correlation with various gene mutation frequencies. This suggests that gene mutation frequency would affect tryptophan metabolism and thus affect prognosis. Tryptophan metabolism-related gene mutations occurred in most LUAD patients, and CNVs play an indispensable role in regulating the transcription level of tryptophan metabolism genes.

By evaluating the infiltration level of different immune cells in the molecular subtypes, we found that the immune infiltration level was the highest in C3 subtype, whereas C1 with the worst prognosis was accompanied by immunosuppression. This suggests that disorders of the tryptophan metabolic pathway would lead to loss of immune function in the TME and thus promote the development of tumor and affect the prognosis of patients. Several studies have shown that IDO1 inhibits T cell response by promoting activation or differentiation of T_reg_ cells [23, 24]. Additionally, kynurenine induces T_reg_ cells by activating aromatic hydrocarbon receptor (AHR), a ligand activated transcription factor that has a great impact on immune cells and participates in the differentiation of T_reg_ cells [25–27]. MDSCs inhibit T cell function, which have strong immunosuppressive activity in the function of CD8^+^ T cells, NK cells, B cells, and other immune cells. In contrast, MDSCs promote tumor angiogenesis and epithelial mesenchymal transition (EMT), secrete matrix metalloproteinases, and differentiate into osteoclasts to promote invasion and metastasis of tumor cells [28, 29]. The scores of MDSCs and T cell exclusion were the lowest in C3, which indicates that C3 had the most active immune function and the least possibility of immune escape. This also means that the immunotherapy strategy would be more effective in C3. This view was further supported by the TIDE score. In contrast, the sensitivity to the four traditional chemotherapeutic drugs was the highest in C1, which indicates that activation of the tryptophan metabolic pathway was related to improving the responsiveness to chemotherapeutic drugs.

The most significantly activated pathway in C1 was G2M checkpoint. It has been reported that the highly active G2M pathway in breast cancer is more invasive and metastatic and is significantly related to the survival rate of patients with breast cancer [30]. However, this pathway was significantly inhibited in C3 subtype, suggesting that tryptophan metabolism genes could promote tumor metastasis by affecting the G2M checkpoint pathway [30]. MYC is a key marker of malignant tumor metastasis. It has been reported that high MYC V1/V2 score is related to malignant cell proliferation and worse clinical and pathological features in metastatic breast cancer. Meanwhile, MYC V1 score is related to high-frequency mutation load. In the mouse lung model of KRAS G12D-driven adenoma, it was found that coactivation of MYC would induce highly proliferative and invasive adenocarcinoma characterized by angiogenic and immunosuppressive stroma [31, 32].

A prognostic risk model was constructed based on five genes, namely, FAM83A, MELTF, CDC25C, ABCC2, and KRT6A. CDC25C directs dephosphorylation of cyclin B-bound CDC2 and triggers mitosis and inhibits p53-induced growth arrest. In addition, CDC25C could directly dephosphorylate CDK1 and activate its kinase activity, indicating that it plays a key role in the regulation of cell division [31]. FAM83A was involved in cell proliferation and epidermal growth factor receptor signaling pathway [33]. Melanotransferrin (MELTF) was identified as a prognostic biomarker in gastric cancer, and the suppression of MELTF reduces the invasion ability of gastric cancer cells [34]. ABCC2 polymorphisms are widely reported to be associated with chemotherapeutic drug response in cancer treatment [35, 36]. KRT6A was reported to promote NSCLC cell growth and invasion through the MYC-regulated pentose phosphate pathway and to promote EMT and cancer stem cell transformation in LUAD [37, 38].

After confirming the robust RS model through the validation set, we found that high RS was often accompanied by worse prognosis, while samples in C3 generally had low RS. The scores of MDSCs, T cell exclusion, and TIDE were higher in the RS-high group than in the RS-low group. This indicates that the RS-high group had obvious immunosuppression and T cell dysfunction, which resulted in immune escape and tumor invasion. At the same time, RS was significantly negatively correlated with the tryptophan metabolism ssGSEA score. This indicates that when tryptophan metabolism is significantly activated, RS significantly decreases, restoring the immune function and improving the prognosis of patients.

## 5. Conclusion

In LUAD, mutation, abnormal expression, or dysfunction of tryptophan metabolism genes leads to abnormality of the tryptophan metabolism pathway. This in turn promotes immunosuppression, immune escape, and occurrence and development of tumors. Ultimately, an abnormal tryptophan metabolism pathway worsens prognosis of LUAD patients.

## Data Availability

The data analyzed in this study could be obtained upon reasonable request.

## Additional Points

*Contribution to the Field Statement*. Of the 40 genes in the tryptophan metabolism pathway, 13 had significant prognostic significance. Based on these 13 genes, three molecular subtypes (C1, C2, and C3) were established. Among them, C1 had the worst prognosis and the lowest enrichment score of tryptophan metabolism. At the same time, C1 had the most genetic variation, the highest level of immune infiltration, and significantly activated pathways related to tumor development. The high-risk and low-risk groups had significant differences in prognosis, immune infiltration, and pathway enrichment, which was consistent with the results of subtype analysis.

## Supplementary Materials

Supplementary 1 Supplement Figure 1: Differences in pathways between the different subtypes. (A) Results of GSEA in different subtypes. (B) Correlation curve between RS and “tryptophan metabolism ssGSEA scores.”

Supplementary 2 Table S1: list of gene sets in the KEGG_TRYPTOPHAN_METABOLISM pathway.
