# Supplementary figures and images for: Correlation of Tryptophan Metabolic Pathway with Immune Activation and Chemosensitivity in Patients with Lung Adenocarcinoma
Source: J Oncol. 2022 Sep 21;2022:2158525. doi: 10.1155/2022/2158525 (PMC9520315; doi:10.1155/2022/2158525)

**A**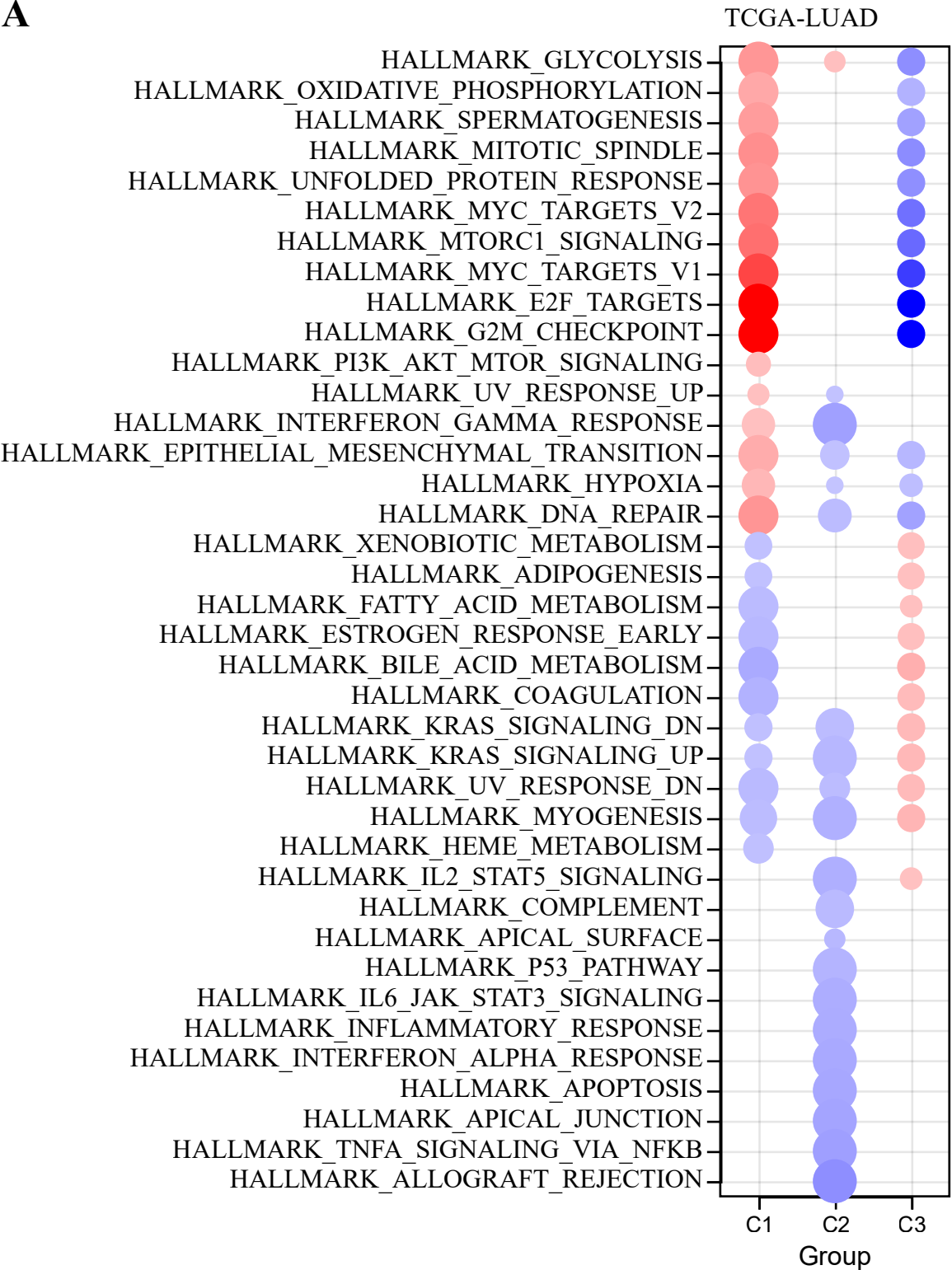**B**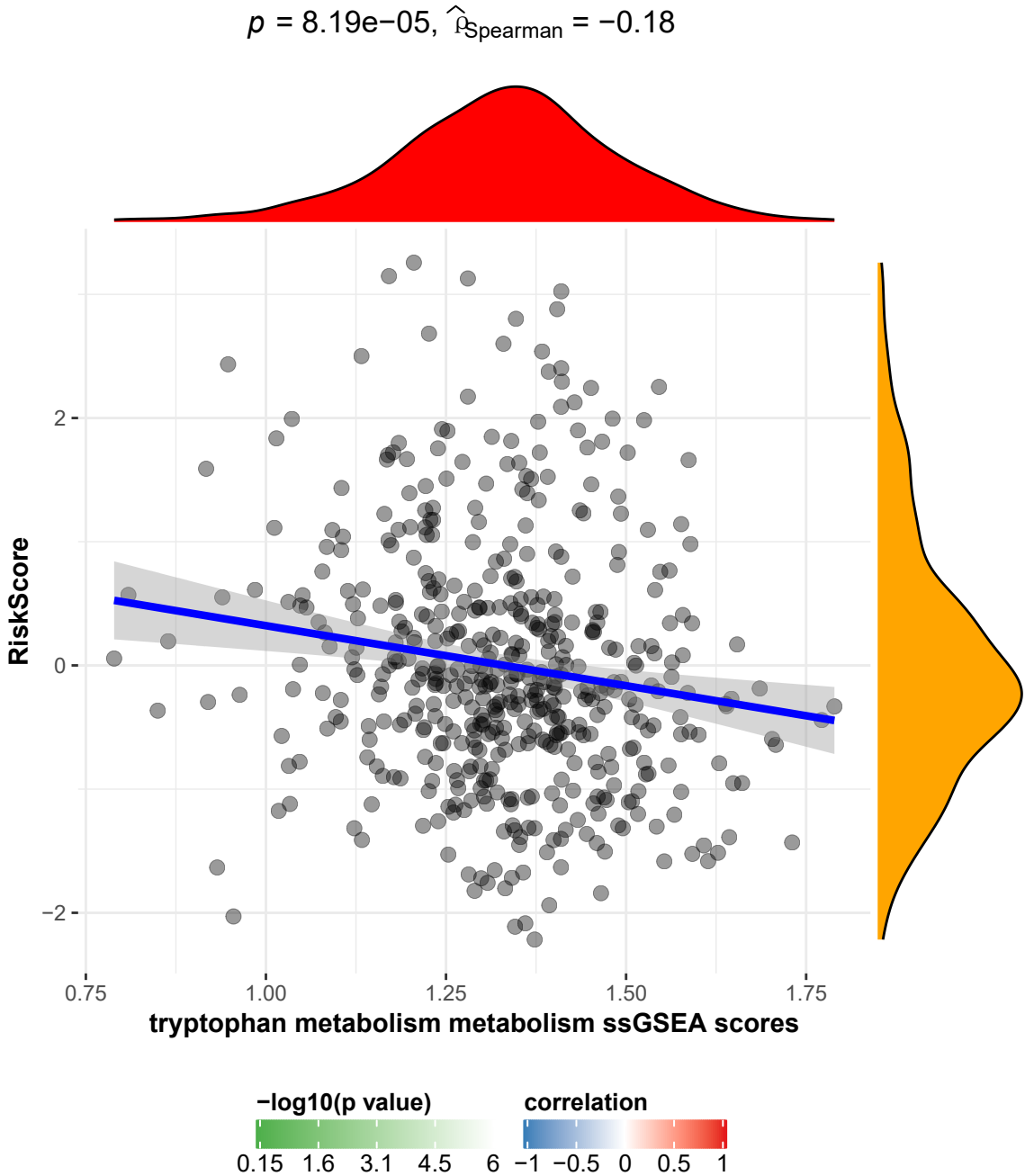

Supplement: Supplementary 1 — Supplement Figure 1: Differences in pathways between the different subtypes. (A) Results of GSEA in different subtypes. (B) Correlation curve between RS and “tryptophan metabolism ssGSEA scores.” [file 2158525.f1.pdf]
