# Supplementary material for: Correlation of Tryptophan Metabolic Pathway with Immune Activation and Chemosensitivity in Patients with Lung Adenocarcinoma
Source: J Oncol. 2022 Sep 21;2022:2158525. doi: 10.1155/2022/2158525 (PMC9520315; doi:10.1155/2022/2158525)
Supplement: Supplementary 2 — Table S1: list of gene sets in the KEGG_TRYPTOPHAN_METABOLISM pathway. [file 2158525.f2.pdf]

genes
AADAT
AANAT
ACAT1
ACAT2
ACMSD
AFMID
ALDH1B1
ALDH2
ALDH3A2
ALDH7A1
ALDH9A1
AOC1
AOX1
ASMT
CAT
CYP1A1
CYP1A2
CYP1B1
DDC
ECHS1
EHHADH
GCDH
HAAO
HADH
HADHA
IDO1
IDO2
IL4I1
INMT
KMO
KYNU
MAOA
MAOB
OGDH
OGDHL
TDO2
TPH1
TPH2
WARS1
WARS2
